# Decreased Soluble Receptor of Advanced Glycation End Product Levels Correlated with Inflammation in Silicosis

**DOI:** 10.1155/2020/2683753

**Published:** 2020-04-14

**Authors:** Heliang Liu, Jingjing Ma, Tian Jiang, Enhong Li, Xiaokun Zhao, Ying Wang, Jie Cui, Xiaohui Hao, Lingli Guo

**Affiliations:** Hebei Key Laboratory of Organ Fibrosis, School of Public Health, North China University of Science and Technology, Tangshan, Hebei 063210, China

## Abstract

Silicosis is a devastating disease caused by inhalation of silica dust that leads to inflammatory cascade and then scarring of the lung tissue. Increasing evidences indicate that soluble receptor for advanced glycation end products (sRAGE) is involved in inflammatory diseases. However, no data on the possible relationship between sRAGE and inflammation of silicosis are available. In this study, serum from subjects with silicosis (*n* = 59) or from healthy controls (HC, *n* = 14) was analyzed for the secretion of sRAGE, tumor necrosis factor-*α* (TNF-*α*), interleukin-1*β* (IL-1*β*), interleukin-6 (IL-6), transforming growth factor-*β*1 (TGF-*β*1), and oxidized low-density lipoprotein (ox-LDL). The associations between sRAGE and cytokines and ox-LDL and lung function were assessed by Pearson's correlation analyses. Mean levels of serum sRAGE were lower in silicosis than those in controls (*p* < 0.05). The subjects who had a longer term of occupational exposure had higher levels of sRAGE (*p* < 0.05). The secretion of TNF-*α*, IL-1*β*, IL-6, TGF-*β*1, and ox-LDL was significantly higher in the silicosis group than that in the HC group (*p* < 0.05). Furthermore, the levels of sRAGE were negatively correlated with TNF-*α*, IL-6, IL-1*β*, and ox-LDL. There is no correlation between sRAGE and TGF-*β*1 and lung function. The optimal point of sRAGE for differentiating silicosis from healthy controls was 14250.02 pg/ml by ROC curve analysis. A decrease in serum sRAGE and its association with inflammatory response might suggest a role for sRAGE in the pathogenesis of silicosis.

## 1. Introduction

Silicosis is one of the most important occupational diseases worldwide [1], while no clinically available therapy is able to revert the progression of the disease effectively [2]. Silicosis is characterized by inflammatory cascade followed by progressive pulmonary fibrosis [3, 4], which leads to respiratory failure due to reduction in gas exchange area and impairment of lung function [5, 6]. Initially, after silica is inhaled, alveolar macrophages (AMs) are activated to release inflammatory cytokines and fibrotic cytokines, such as tumor necrosis factor-*α* (TNF-*α*), interleukin-1*β* (IL-1*β*), interleukin-6 (IL-6), and transforming growth factor-*β*1 (TGF-*β*1) [7–10], which play crucial roles in inflammatory response and fibrosis of silicosis. Oxidized low-density lipoprotein (ox-LDL) is considered as a marker of inflammation [11] and oxidative stress [12, 13] in several inflammatory diseases. Furthermore, our previous study found that the levels of ox-LDL are increased in alveolar macrophages of patients with silicosis [14], suggesting a possible role of ox-LDL in inflammation of silicosis.

Receptor for advanced glycation end products (RAGE), a pattern-recognition receptor, has been reported to amplify or sustain immune and inflammatory responses [15–17] and drive fibrotic process [18]. Soluble receptor for advanced glycation end products (sRAGE) is the extracellular form of RAGE and is produced either by proteolytic cleavage of RAGE or through alternative RNA splicing [19–21]. sRAGE prevents the interaction between RAGE and ligands by acting as a decoy receptor [22]. Studies have found that sRAGE have a protective effect against inflammation through inhibiting RAGE signaling [23, 24]. And sRAGE has been recognized as a biomarker or therapeutic target in inflammatory diseases [22, 25–27] and fibrotic diseases [28], while its role in silicosis remains obscure. Based on the above questions, we explored the relationship between sRAGE and silicosis in this study.

## 2. Materials and Methods

### 2.1. Study Subjects

59 patients with silicosis (12 stage I of silicosis (SI), 17 stage II of silicosis (SII), and 30 stage III of silicosis (SIII), respectively) who underwent massive whole lung lavage at Beidaihe Chinese coal workers nursing home and 14 healthy subjects who worked in an iron mine of Henan province were enrolled in this study. The diagnosis of silicosis was done by clinical and radiological findings on high-quality X-ray according to the diagnostic criteria of pneumoconiosis (GBZ 70-2009, China). The criteria describe silicosis as stages 0, I, II, and III. In this study, patients at stage 0 were not included. Patients were excluded if they met any of the following criteria: (1) other inflammatory diseases; (2) other fibrotic diseases; (3) other pulmonary diseases, such as COPD, active tuberculosis, pneumonia, and pulmonary heart disease; (4) autoimmune disorders; and others. The parameters of lung function were measured by the Puritan Bennett™ 840 Ventilator. All participants provided written informed consent. All procedures performed were in accordance with the 1964 Helsinki declaration and its later amendments or comparable ethical standards, and ethical approval was obtained from the Clinical Trial and Ethics Committee of North China University of Science and Technology.

### 2.2. Measurement for sRAGE, TNF-*α*, IL-1*β*, IL-6, TGF-*β*1, and ox-LDL

Peripheral blood was collected. Serum was separated by centrifugation for 10-15 min at 3000 rpm and stored at −80°C for analysis. The secretion of sRAGE, TNF-*α*, IL-1*β*, IL-6, TGF-*β*1, and ox-LDL in serum was detected by ELISA assay (sRAGE, IL-6, IL-1*β*, and TGF-*β*1 ELISA kit, BOSTE, Wuhan, Hubei; TNF-*α* ELISA kit, eBioscience, San Diego, CA; ox-LDL ELISA kit, Nanjing Xinfan Biology, Nanjing, China). All measurements were carried out strictly according to the manufacturer's instructions.

## 3. Statistical Analysis

Results are presented as mean ± standard deviation (SD) for normally or median (25th, 75th percentile) for nonnormally distributed data. Differences between groups were statistically analyzed using Student's *t*-test or one-way ANOVA tests for normally distributed data, the Mann-Whitney *U* test for nonnormally distributed data, and the Chi-square test for categorical variables. Correlations of sRAGE with cytokines, ox-LDL, and lung function were performed using Pearson's correlation test. Receiver operating characteristic (ROC) curve analysis was applied to test the association of sRAGE levels at baseline with disease outcome. The threshold of significance was set at 5%. Data were analyzed using SPSS 17.0 for Windows.

## 4. Results

### 4.1. Clinical Characteristics of Subjects

Table 1 shows the characteristics of subjects involved in this study. Sex ratio, age, smoking pack-year, years of occupational exposure to silica dust, and BMI did not differ between groups. FEV_1_% pre, FVC% pre, and FEV_1_/FVC in silicosis were significantly lower than healthy controls suggesting a significant decline in lung function of patients with silicosis.

### 4.2. Serum Levels of sRAGE, TNF-*α*, IL-1*β*, IL-6, TGF-*β*1, and ox-LDL

**Figure 1:**
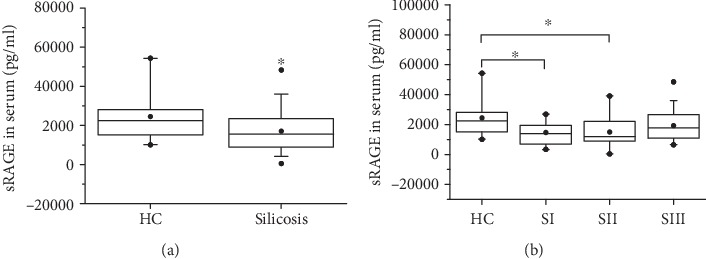
Serum levels of sRAGE in subjects. The secretion of sRAGE in serum was detected by ELISA. HC: healthy controls; SI: stage I of silicosis; SII: stage II of silicosis; SIII: stage III of silicosis. ^∗^*p* < 0.05.

**Figure 2:**
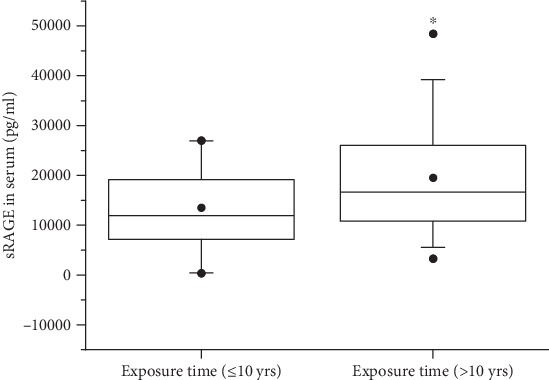
Serum levels of sRAGE in patients with silicosis. The secretion of sRAGE in serum was detected by ELISA. ^∗^*p* < 0.05.

Since circulating sRAGE may be a biomarker during chronic inflammation, we tested sRAGE levels by ELISA in serum from patients with silicosis in this study. As shown in Figure 1(a), the levels of sRAGE in the silicosis group were significantly lower than those in the control group. More specifically, mean serum levels of sRAGE in SI (14799.00 ± 757.43 pg/ml) and SII (15117.00 ± 10039.00 pg/ml) were lower than those in HC (24411.12 ± 11408.58 pg/ml). The secretion of sRAGE in the SIII group was lower than that in the HC group, but there was no statistical difference between two groups (Figure 1(b)). Moreover, the concentration of sRAGE in patients with silicosis who had worked for a few years (≤10 years) was lower than those who had worked for a longer period (>10 years), suggesting that the duration of silica stimulation contributes to the levels of sRAGE (Figure 2).

**Figure 3:**
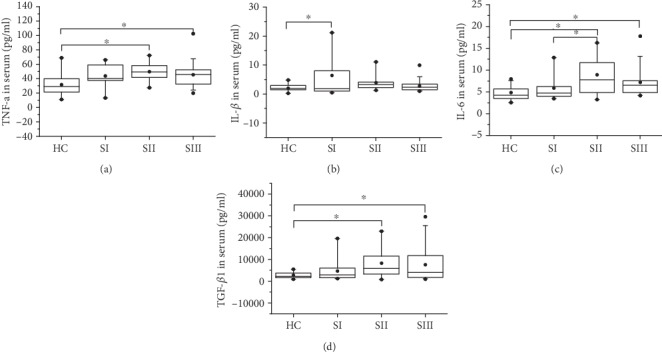
Serum levels of TNF-*α*, IL-1*β*, IL-6, and TGF-*β*1. The secretion of (a) TNF-*α*, (b) IL-1*β*, (c) IL-6, and (d) TGF-*β*1 in serum was detected by ELISA analysis. SI: stage I of silicosis; SII: stage II of silicosis; SIII: stage III of silicosis. ^∗^*p* < 0.05.

Next, we measured the levels of cytokines TNF-*α*, IL-1*β*, IL-6, and TGF-*β*1 and inflammation marker ox-LDL in serum. As shown in Table 2, the secretion of TNF-*α*, IL-6, TGF-*β*1, and ox-LDL was increased in patients with silicosis in comparison to controls, except for IL-1*β*. Further investigation found that the secretion of IL-1*β* was increased in the SI group compared with that in the HC group (2.05 ± 1.28 pg/ml vs. 6.38 ± 7.86 pg/ml) (Figure 3(b)). The levels of TNF-*α* in the SII (49.47 ± 13.49 pg/ml) and SIII groups (45.62 ± 16.58 pg/ml) were higher than those in the HC group (31.85 ± 16.72 pg/ml) (Figure 3(a)). The concentration of IL-6 in the SII (8.91 ± 4.26 pg/ml) and SIII (6.96 ± 2.96 pg/ml) groups was higher than that in the HC group (4.67 ± 1.79 pg/ml). And the levels of IL-6 in the SII group were increased compared with that in the SI group (Figure 3(c)). Patients in the SII and SIII groups showed significantly increased levels of TGF-*β*1 as compared to healthy controls (83145.44 ± 73720.2 pg/ml, 75596.33 ± 74498.49 pg/ml vs. 27018.27 ± 13759.52 pg/ml, respectively) (Figure 3(d)).

### 4.3. Correlations between sRAGE and Cytokines and ox-LDL and Lung Function

Serum levels of sRAGE in those subjects correlated inversely with the levels of TNF-*α* (*r* = −0.241, *p* = 0.049), IL-1*β* (*r* = −0.288, *p* = 0.028), IL-6 (*r* = −0.413, *p* = 0.001), and ox-LDL (*r* = −0.283, *p* = 0.035) (Table 3). However, no correlation between sRAGE and TGF-*β*1, and also lung function parameters, such as FEV_1_, FVC, FVC% pre, FEV_1_% pre, and FEV_1_/FVC, were found (Table 3).

### 4.4. The Cut-Off Point of sRAGE Determined by ROC Analysis

**Figure 4:**
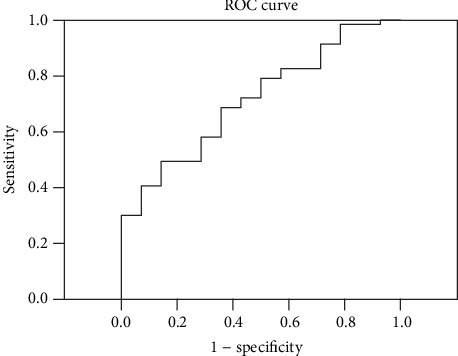
Receiver operating characteristic (ROC) curve for sRAGE (AUC = 0.713) to discriminate between silicosis patients and healthy controls.

Using ROC analysis, we found that 14250.02 pg/ml was the best serum sRAGE cut-off level (sensitivity 49.1% and specificity 85.7%, respectively) to distinguish between healthy controls and silicosis patients (AUC = 0.713) (Figure 4).

## 5. Discussion

In the present study, we found the levels of sRAGE in patients with silicosis were lower than those in healthy controls. And the levels of TNF-*α*, IL-6, IL-1*β*, TGF-*β*1, and ox-LDL were increased in silica-exposed subjects. Correlational analysis showed that the levels of sRAGE were negatively correlated with TNF-*α*, IL-6, IL-1*β*, and ox-LDL. However, there was no correlation between sRAGE and TGF-*β*1, and also lung function. These findings suggested that sRAGE may be involved in the pathogenesis of inflammation of silicosis.

sRAGE acts as a decoy of RAGE signaling, thereby inhibiting the interaction between RAGE and proinflammatory ligands (such as HMGB1 and AGEs), which have been proposed to play critical roles in multiple inflammatory diseases [23, 29, 30]. Low sRAGE levels have been reported in studies of individuals with inflammatory lung diseases, such as COPD [25, 31], asthma [32], and cystic fibrosis [33]. Consistent with these findings, we found that serum levels of sRAGE were decreased in patients with silicosis. Further investigation observed that sRAGE levels were reduced in SI and SII, while there was no significant decline in patients with SIII. It is important to note, however, in our another work, we found that the levels of sRAGE in BALF of patients with SIII, but not SI and SII, were lower than those in healthy controls. The different subjects involved in the control group might be an important factor contributing to the inconsistent results obtained from BALF and serum. In specific, the control subjects in this study were healthy individuals, while the control subjects in our previous study were observation individuals who had a history of silica exposure, but X-ray results did not meet the standard of stage I silicosis. Moreover, we found the levels of sRAGE in patients with silicosis who had worked for a few years (≤10 years) were lower than those who had worked for a longer period (>10 years). We speculated that the longer silica exposure time might produce more inflammatory mediators in the lung. Subsequently, more anti-inflammatory mediators, such as sRAGE, would be secreted to maintain inflammatory-anti-inflammatory dynamic circumstance. Therefore, the longer occupational exposure duration might induce the higher levels of sRAGE in silicosis.

Silicosis is a complicated disease, which can present as acute silicosis, accelerated silicosis, and chronic silicosis [34]. As far as we know, the diagnosis of silicosis is based on a history of silica exposure and the radiography abnormalities. Although the association between the reduction of lung function and silicosis was replicated in several studies [35], lung function loss in the absence of silicosis would occur until between 30 and 40 years of silica exposure. Nevertheless, the lung function test is an important method to estimate pulmonary function impairment or figure out the type of respiratory function abnormalities (obstructive? or restrictive? or mixed?) in silicosis [34]. In the present study, we measured the lung function of individuals to investigate the possible relationship between lung function loss and silicosis. We focus on FEV_1_ and FVC, which were usually used to evaluate lung function loss in silicosis [35–38]. We found that FVC% pre, FEV_1_% pre, and FEV_1_/FVC (%) were decreased in the silicosis group. It is in agreement with similar observation in other works, where lung function significantly declined in patients with silicosis [39, 40].

Recent studies demonstrated that serum sRAGE were positively correlated with FEV_1_ and FEV_1_/FVC in COPD subjects [41] and correlated with FVC and DLCO in patients with IPF [42]. However, in the current study, there was no correlation between sRAGE and lung function in silicosis. Similar to our results, Lyu et al. also found no correlation between sRAGE and lung function in patients with asthma [43]. The possible reason may be as follows: silicosis is mainly characterized by inflammation at the early stage and followed by fibrosis at the later stage. Patients may have no pulmonary function abnormalities in the early stage. The decline in lung function mainly happened at the fibrotic stage in silicosis. Our findings observed that sRAGE were negatively correlated with TNF-*α*, IL-6, IL-1*β*, and ox-LDL (indicators of inflammation), but not TGF-*β*1 (indicator of fibrosis), suggesting that sRAGE might be an anti-inflammatory and not an antifibrotic marker in silicosis. Therefore, the critical role of sRAGE in inflammation of silicosis may lead to no correlation between sRAGE and lung function.

It is well established that ox-LDL is involved in multiple inflammatory diseases, such as cardiovascular disease [44] and obesity [45], mainly by activating both innate and adaptive immunity. Our previous study showed that ox-LDL were increased in AMs of silicosis [14], implying a critical role of ox-LDL in inflammation of silicosis. In the present study, we observed that serum levels of ox-LDL were significantly enhanced in patients with silicosis. Moreover, the levels of ox-LDL were negatively correlated with levels of sRAGE in silicosis. Similar to our findings, Kotani et al. reported that circulating sRAGE was inversely correlated to ox-LDL in serum of asymptomatic subjects [46]. Those studies mentioned above suggested a close relationship between sRAGE and ox-LDL. It has been found that RAGE mediates the ox-LDL-induced activation of MAPK signaling in osteoblastic differentiation process [47] and production of ROS as well as cell–cell adhesion in endothelial cells [48], indicating that RAGE might be considered as a target receptor for ox-LDL [49]. Accordingly, we speculated that decreased sRAGE might lead to the activation of ox-LDL/RAGE signaling, subsequently increasing the levels of ox-LDL in silicosis.

Our study had two limitations. First, the sample size was small in groups, especially in the healthy control group. Second, we did not demonstrate the detailed molecular mechanisms of sRAGE in silicosis. Hence, enlarged sample size and mechanism study are necessary in the future. Nevertheless, our findings observed an association between sRAGE and silicosis, which should help us to understand the possible mechanisms responsible for inflammation of silicosis.

## 6. Conclusions

Our study showed that serum sRAGE levels were decreased in silicosis. The secretion of sRAGE was correlated with inflammation in silicosis. Clinically, serum sRAGE may be a promising intervention target in silicosis, while the role of sRAGE needs to be further clarified.

## Figures and Tables

**Table 1:** Clinical characteristics of study subjects.

Clinical characteristics	HC (*n* = 14)	Silicosis (*n* = 59)	*p* value
Male/female	13/1	58/1	0.349^a^
Age (yrs)	42.94 ± 10.42	47.34 ± 6.32	0.097^b^
Smoking pack-years (yrs)	20.0 (10.0, 20.0)	20.0 (9.4, 21.25)	0.981^c^
Years of occupational exposure to silica dust (yrs)		15.13 ± 7.66	—
BMI	23.67 ± 3.40	19.41 ± 7.85	0.288^b^
FVC (L)	3.32 ± 0.42	3.18 ± 0.81	0.676^b^
FVC% pre	87.3 ± 8.16	70.18 ± 15.34	**0.033** ^b^
FEV_1_ (L)	2.78 ± 0.37	2.27 ± 0.84	0.145^b^
FEV_1_% pre	81.88 ± 8.48	59.11 ± 20.50	**0.033** ^b^
FEV_1_/FVC (%)	80.22 ± 1.10	69.30 ± 15.88	**0.0001^b^**

BMI: body mass index; FEV_1_: forced expiratory volume in 1 s; FVC: forced volume capacity; FVC% pre: FVC% predicted; FEV_1_% pre: FEV1% predicted. Values are mean ± SD for normally distributed data or median (75th, 25th percentile) for nonnormally distributed data. ^a^The differences were calculated by the Chi-square test. ^b^The differences were calculated by Student's *t*-tests. ^c^The differences were calculated by the Mann-Whitney *U* test.

**Table 2:** The secretion of cytokines and ox-LDL (pg/ml).

	HC (*n* = 14)	Silicosis (*n* = 59)	*p* value^a^
TNF-*α*	31.85 ± 16.72	46.46 ± 15.34	**0.004**
IL-1*β*	2.052 ± 1.28	3.65 ± 3.84	0.181
IL-6	4.67 ± 1.79	7.39 ± 3.48	**0.002**
TGF-*β*1	27018.27 ± 13759.52	73475.64 ± 73416.33	**0.042**
ox-LDL	10.56 ± 10.98	19.98 ± 11.98	**0.026**

^a^The differences were calculated by Student's *t*-test.

**Table 3:** The correlations between sRAGE and cytokines and ox-LDL and lung function.

	sRAGE	
	*r*	*p* value^a^
TNF-*α*	-0.241	**0.049**
IL-1*β*	-0.288	**0.028**
IL-6	-0.413	**0.001**
TGF-*β*1	-0.096	0.452
ox-LDL	-0.283	**0.035**
FVC	0.056	0.673
FEV_1_	0.099	0.456
FVC% pre	0.154	0.262
FEV_1_% pre	0.042	0.759
FEV_1_/FVC	-0.051	0.710

^a^The correlation between sRAGE and parameters was calculated by Pearson's correlation test.

## Data Availability

The data used to support the findings of this study are available from the corresponding author upon request.
